# Construction of an immune-related risk score signature for gastric cancer based on multi-omics data

**DOI:** 10.1038/s41598-024-52087-3

**Published:** 2024-01-16

**Authors:** Ying Wang, Wenting Huang, Shanshan Zheng, Liming Wang, Lili Zhang, Xiaojuan Pei

**Affiliations:** 1 Department of Oncology, National Cancer Center/National Clinical Research Center for Cancer/Cancer Hospital and Shenzhen Hospital, Chinese Academy of Medical Science and Peking Union Medical College, Shenzhen, Guangdong China; 2 Department of Pathology, National Cancer Center/National Clinical Research Center for Cancer/Cancer Hospital and Shenzhen Hospital, Chinese Academy of Medical Science and Peking Union Medical College, Shenzhen, Guangdong China; 3 Department of Gastrointestinal Surgery, National Cancer Center/National Clinical Research Center for Cancer/Cancer Hospital and Shenzhen Hospital, Chinese Academy of Medical Science and Peking Union Medical College, Shenzhen, Guangdong China; 4 Department of Pathology, Shenzhen Hospital, Southern Medical University, Shenzhen, Guangdong China

**Keywords:** Cancer, Gastrointestinal cancer, Tumour biomarkers

## Abstract

Early identification of gastric cancer (GC) is associated with a superior survival rate compared to advanced GC. However, the poor specificity and sensitivity of traditional biomarkers suggest the importance of identifying more effective biomarkers. This study aimed to identify novel biomarkers for the prognosis of GC and construct a risk score (RS) signature based on these biomarkers, with to validation of its predictive performance. We used multi-omics data from The Cancer Genome Atlas to analyze the significance of differences in each omics data and combined the data using Fisher's method. Hub genes were subsequently subjected to univariate Cox and LASSO regression analyses and used to construct the RS signature. The RS of each patient was calculated, and the patients were divided into two subgroups according to the RS. The RS signature was validated in two independent datasets from the Gene Expression Omnibus and subsequent analyses were subsequently conducted. Five immune-related genes strongly linked to the prognosis of GC patients were obtained, namely CGB5, SLC10A2, THPO, PDGFRB, and APOD. The results revealed significant differences in overall survival between the two subgroups (*p* < 0.001) and indicated the high accuracy of the RS signature. When validated in two independent datasets, the results were consistent with those in the training dataset (*p* = 0.003 and *p* = 0.001). Subsequent analyses revealed that the RS signature is independent and has broad applicability among various GC subtypes. In conclusion, we used multi-omics data to obtain five immune-related genes comprising the RS signature, which can independently and effectively predict the prognosis of GC patients with high accuracy.

## Introduction

Early identification of stomach cancer is associated with a superior survival rate compared to that of advanced gastric cancer (GC)^1–4^. As traditional biomarkers are not effective at predicting the prognosis of GC patients^5,6^, novel therapeutic biomarkers are of crucial for improving prognoses.

The use of multi-omics data has the ability to uncover deeper insights^7^. Several recent studies have demonstrated that multi-omics data can be used to identify novel biomarkers for early diagnosis and treatment from new perspectives^8–11^ and that these biomarkers can improve the prognosis of cancer patients. Fisher’s method is considered a classical method that can integrate information from multiple omics into one feature^12,13^. A multi-omics study based on the TCGA database was performed. We utilized RNA sequencing (RNA-Seq) expression, copy number variation (CNV) and DNA methylation data, and tests for each OMC revealed distinct characteristics of the marker genotype^14^. The Fisher test was used to combine the p-values of each OMC, from which we combined the information from multiple views to screen for GC-associated genetic markers.

Tumor-infiltrating immune cells have been demonstrated to be linked to promoting and preventing cancer progression in distinct cancer types^15,16^. Immune checkpoints are a class of components that are upregulated in the TME and inhibit antitumor T-cell responses^17^. Classifying the influence of genetic markers on the prognosis and diagnosis of tumor-infiltrating immune cells and immunological checkpoints might improve the treatment and survival of GC patients.

In this study, five immune-related genes related to GC patient prognosis that can serve as prospective biomarkers were found from multi-omics data in the TCGA database and utilized to construct a risk score (RS) signature for each patient and establish an RS signature. The results suggested that the RS signature created in this work accurately predicts the prognostic outcome of GC patients with greater predictive power than standard clinical indicators when validated using the Gene Expression Omnibus (GEO) database.

## Materials and methods

### Data preparation

**Figure 1:**
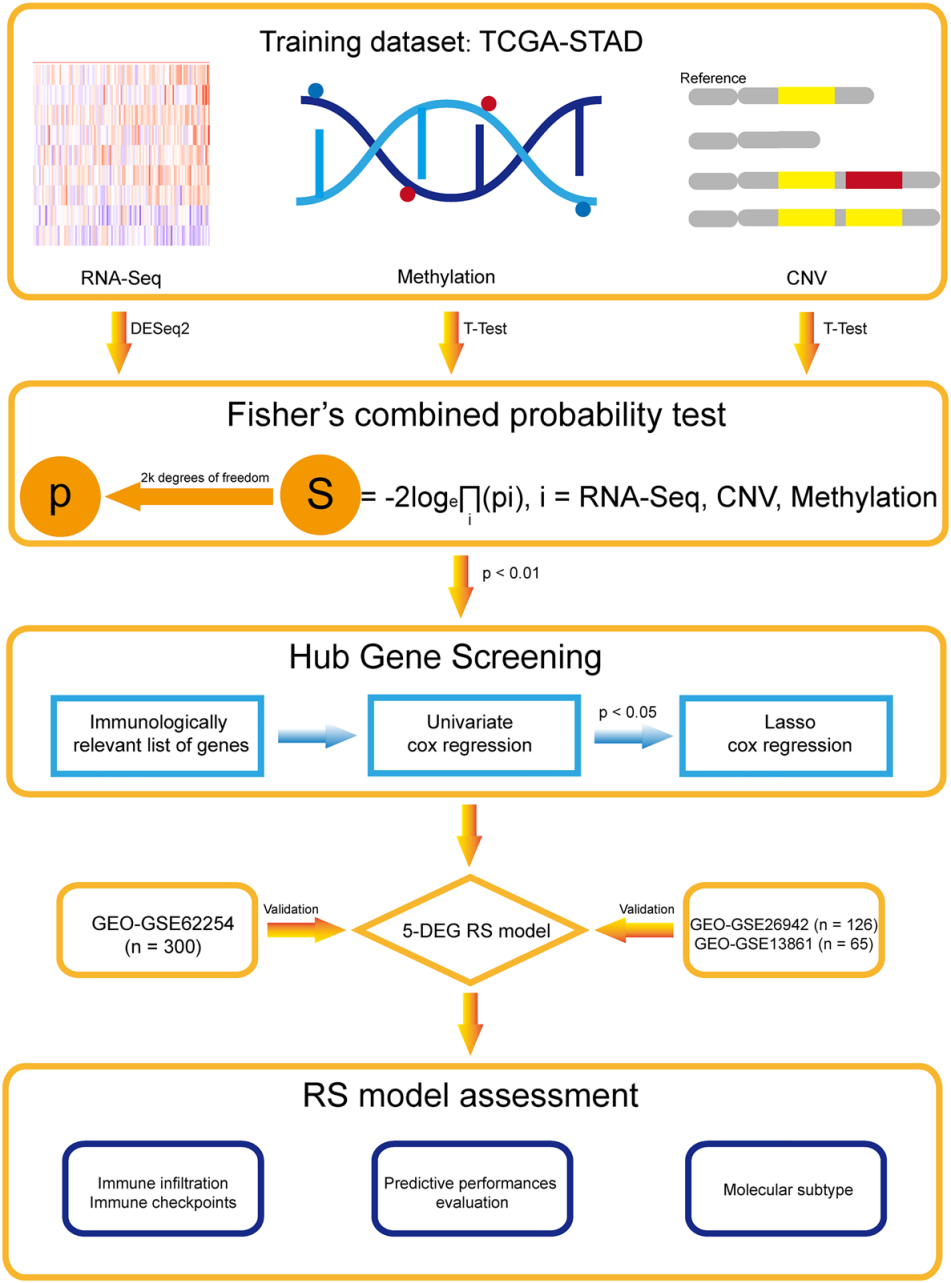
Workflow of this study.

This study followed the workflow shown in Fig. 1. The University of California Santa Cruz (UCSC) database (https://xenabrowser.net/datapages/) was used to obtain gene expression RNA-Seq data (n = 442), DNA methylation 27k array data (n = 142), gene-level CNV data (n = 413), and clinical information. GSE62254 (n = 300), GSE26942 (n = 126), and GSE13861 (n = 65) RNA-Seq data, as well as corresponding patient clinical information, were downloaded from the GEO (http://www.ncbi.nlm.nih.gov/geo/). The present study used TCGA datasets for training. The GSE62254 dataset served as the first independent validation dataset. Due to the limited sample size, GSE13861 was combined with GSE26942 to create a second independent validation dataset (n = 191).

### Hub gene screening

All the data analyses were performed in R (version 4.1.2). First, RNA-Seq data, methylation 27k array data, and CNV data from the TCGA were filtered to retain only genes present in all three datasets (11,246 genes in total). The DESeq2^18^ package (version 1.34.0) was used to calculate *p* values (*p*_*RNAs*_) for the significance of differential expression between tumor samples (n = 389) and normal tissue samples (n = 33) for each gene in the training dataset. Similarly, *p*-values (*p*_*methy*_) were calculated for each gene in methylation 27 k array data between tumor samples and normal tissue samples using the Student's t test^19^. On the basis of CNV data, patients in the training dataset were divided into copy number variation and nonvariation groups. The *p*-values (*p*_*CNVs*_) of the DEGs between these two groups in the RNA-Seq dataset were subsequently calculated using Student's t test. After obtaining the *p* value for each gene in the three omics analyses, we calculated the S statistic using the Fisher's method^13^(1); three independent *p*-values with 2k degrees of freedom were then used to transform the S statistic into the null hypothesis *p* value (*p*_*combined*_). The *p*_*combined*_ value was considered to represent the significance of the gene for the prognostic profile of GC patients according multi-omics to data. Genes with a *p*_*combined*_ value less than 0.010 were considered to be significant.

1 $${\text{S}}= -2{{\text{log}}}_{e}\prod_{i}p\left(i\right)$$

In Eq. (1), i represents the *p*_*RNA*_, *p*_*methy*_, or *p*_*CNV*_ of each gene. Using the IMMPORT database (http://www.immport.org), we downloaded a list of immune-related genes (IRGs) and retained only significant IRGs. The remaining genes in the RNA-Seq and methylation data combined with clinical information were subsequently analyzed via univariate Cox regression analysis. Genes with a *p* value less than 0.050 in both sets of results were considered significant. Further screening was then performed using LASSO regression analysis, the results of which revealed candidate genes that correlated strongly with the prognosis of GC patients. The pheatmap^20^ package (version 1.0.12) was used to plot heatmaps showing differences in candidate gene expression between tumor and normal tissue samples.

### Functional enrichment analysis

Gene Ontology (GO) and Kyoto Encyclopedia of Genes and Genomes (KEGG) enrichment analyses were carried out on the genes screened out from multi-omics data to explore molecular mechanisms by the “clusterProfiler”^21^ R package (version 4.2.2) and the “org.Hs.eg.db”^22^ R package (version 3.14.0). *p* values were adjusted using the *fdr* method to control the false discovery rate (FDR).

### RS signature development and validation

First, the risk score of each patient in the training dataset was calculated using Eq. (2).

2 $$Risk \; score= \sum \beta RNA*ExpRNA$$

β_RNA_ represents the coefficient of the candidate gene according to the univariate Cox regression analysis of the RNA-Seq data, and Exp_RNA_ represents expression of the candidate gene in RNA-Seq data. Patients were divided into high risk and low risk subgroups based on the median score.

We first observed the distribution of each clinical indicator in the different subgroups and assessed whether the distribution was significantly different using the chi-square test or Fisher's exact test. Furthermore, the survival (version 3.3.1)^23^ and survminer (version 0.4.9)^24^ packages were used to perform survival analysis on the high and low risk subgroups of the training dataset to analyze differences in overall survival (OS) between the two subgroups; the results of survival analysis were generated using Kaplan–Meier (KM) survival curves. To assess the accuracy of the RS in predicting the prognosis of GC patients, the survivalROC package^25^ (version 1.0.3) was used to plot receiver operating characteristic (ROC) curves, and the area under the ROC curve (AUC) was used to determine the predictive accuracy of the RS signature. A similar approach was used for survival and ROC analyses for the two independent validation datasets from GEO cohort divided into high and low risk subgroups.

### RS signature assessment

To explore the prognostic predictive capability of the RS signature in patients with GC in different subgroups, we performed survival and ROC analyses using the same approach as above for two subgroups of patients with stages I & II and III & IV in the TCGA training dataset. In 2014, TCGA classified gastric cancer patients into four molecular subtypes: Epstein–Barr virus (EBV) positive, microsatellite unstable (MSI), genomically stable (GS), and chromosomal unstable (CIN)^26^. For the training dataset, violin plots were generated to illustrate differences in distributions of RSs among the four subtypes, and survival analysis was performed for each subtype. Afterward, univariate and multivariate Cox regression analyses were conducted to verify the superior predictive power of the RS signature over traditional clinical prognostic indicators. To assess the independence and predictive power of the RS signature, it was used as a prognostic indicator for GC patients in the training dataset in univariate and multivariate Cox regression analyses with other clinical indicators (age, sex, and stage). Moreover, a nomogram was drawn to better represent the predictive power of the RS and other clinical indicators.

### Immune characteristics

To explore differences in immune characteristics between patients in the high and low risk subgroups, the CIBERSORT^27^ algorithm and the LM22 gene signature were used to analyze differences in immune infiltration between patients in the high and low risk subgroups in the TCGA training dataset and the GSE62254 independent validation dataset. In the next step, we analyzed the differences in expression of 33 immune checkpoint molecules (Supplementary file) in the TCGA training cohort to investigate differences between immune mechanisms in high and low risk subgroups.

### Molecular docking

By combining molecular docking analyses, we aim to comprehensively explore ligand-target interactions, ultimately advancing our understanding of molecular mechanisms and informing the development of novel therapeutic agents. Drugs that differed significantly between the high and low risk groups were first identified by calculating half maximal inhibitory concentration (IC50) values, after which the molecular operating environment (MOE) was used to predict interactions of the five constituent modeled genes with these drugs.

## Results

### Identification of prognostic genes in the TCGA dataset with multi-omics data

**Figure 2:**
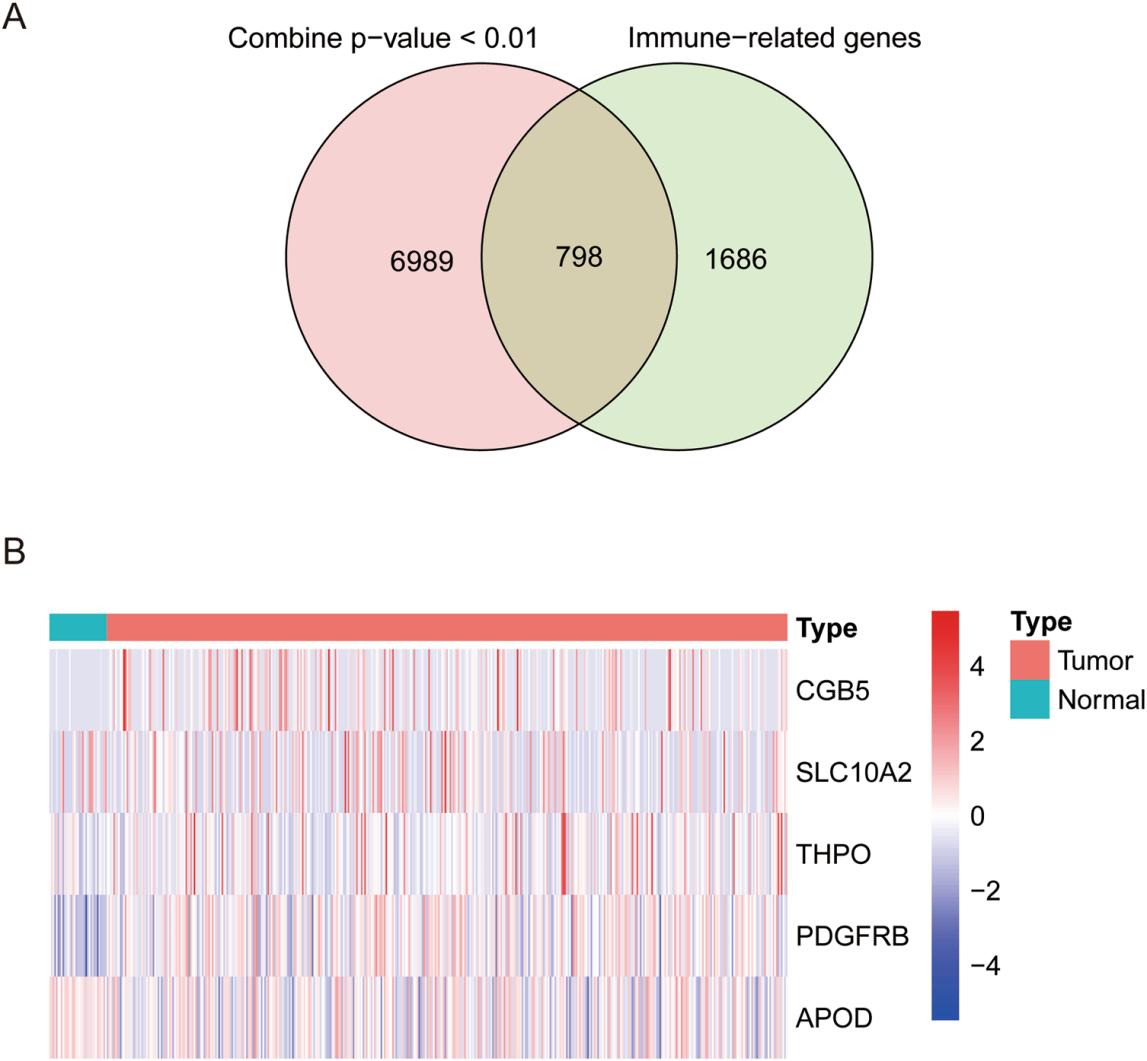
Identification of 5 prognostic genes. (**A**) Venn diagram showing 798 overlapping genes between genes screened out by multi-omics data and immune-related genes. (**B**) Heatmap showing differences in expression of the 5 prognostic genes between tumor and normal tissues.

Based on *p*_*combined*_ calculated using multi-omics data from the training dataset, 7787 genes were screened out as *p*_*combined*_ < 0.010, 798 of which are IRGs (Fig. 2A). Among these IRGs, 16 genes associated with GC prognosis were identified through univariate Cox analysis of RNA-Seq and DNA methylation data from the training dataset. Five genes were subsequently selected by LASSO regression; CGB5, THPO, and PDGFRB were upregulated in the tumor tissue, while SLC10A2 and APOD were downregulated (Fig. 2B). These 5 genes correlated positively with poor prognosis in GC patients (Table 1).

**Table 1:** Univariate Cox analysis of associations between five hub genes and OS in the TCGA dataset.

Gene symbol	Coefficient	Hazard ratio	p-value
CGB5	0.157	1.170	< 0.001
SLC10A2	0.077	1.080	0.025
THPO	0.112	1.119	0.002
PDGFRB	0.199	1.220	0.005
APOD	0.129	1.138	< 0.001

### Construction of an RS signature using the TCGA dataset

**Figure 3:**
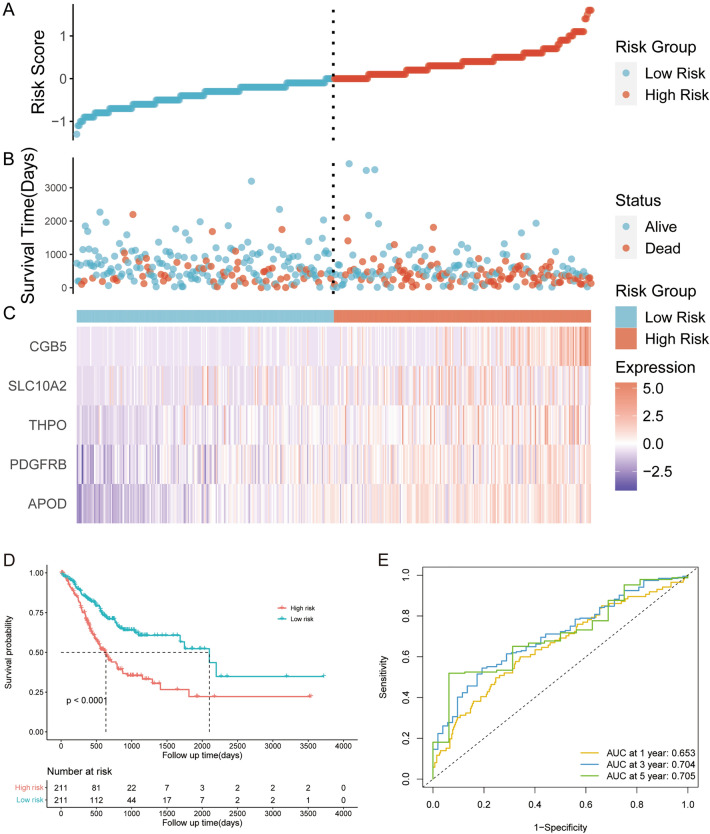
The prognostic value of the 5-genes RS signature in the training dataset. (**A**) The distribution of risk scores in the training dataset. (**B**) The scatter plot of the 5-genes RS signature distribution for patient survival status in the training dataset. (**C**) Expression of the 5 prognostic genes in patients with different RSs in the training dataset. (**D**) Survival analysis of OS in patients with different RSs in the training dataset.

Through Cox and LASSO regression analyses, the 5 hub genes (Table 1) that most contributed to the OS of GC patients were screened out and used to construct an RS signature with the following formula (Formula 2): Risk score = (0.157 × Exp_CGB5_) + (0.077 × Exp_SLC10A2_) + (0.112 × Exp_THPO_) + (0.199 × Exp_PDGFRB_) + (0.129 × Exp_APOD_). The risk score of each patient was calculated, and patients in the TCGA training dataset were divided into two subgroups: high risk (n = 211) and low risk (n = 211), using the median score as the cutoff value. As shown in Fig. 3A–C, patients with high risk scores had higher mortality rates and expression of the 5 immune-related genes. KM survival analysis was subsequently performed to evaluate the effect of the RS signature on the OS of patients with GC in the training dataset (Fig. 3D). The results indicated that patients in the high risk subgroup had significantly poorer prognosis than did those in the low risk subgroup (*p* < 0.001). Time-dependent ROC analysis was further performed to assess the predictive performance of the RS signature. As presented in Fig. 3E, the AUC reached 0.653 at 1 year, 0.704 at 3 years, and 0.704 at 5 years, demonstrating the prognostic value of the RS signature.

Table 2 shows the distributions of clinical characteristics among patients in the high risk and low risk subgroups. The distributions of patients according to American Joint Committee on Cancer (AJCC) TNM stage and tumor status were significantly different between the high and low risk subgroups.

**Table 2:** Clinical characteristics of the high risk and low risk groups.

Clinical indicators^a^	High risk (n = 211)	Low risk (n = 211)	p value for chi-square tests^b^
Age			0.254
≥ 65 years	113 (53.6)	125 (59.2)	
< 65 years	97 (46.0)	84 (39.8)	
Missing	1 (0.5)	2 (0.9)	
Sex			1.000
Male	138 (65.4)	137 (64.9)	
Female	73 (34.6)	74 (35.1)	
AJCC TNM stage			0.011
Stage I	18 (8.5)	39 (18.5)	
Stage II	67 (31.8)	70 (33.2)	
Stage III	91 (43.1)	82 (38.9)	
Stage IV	26 (12.3)	15 (7.1)	
Stage x or missing	9 (4.3)	5 (2.4)	
T			0.011
T1	5 (2.4)	17 (8.1)	
T2	42 (19.9)	52 (24.6)	
T3	95 (45.0)	95 (45.0)	
T4	66 (31.3)	46 (21.8)	
Tx or missing	3 (1.4)	1 (0.5)	
N			0.132
N0	52 (24.6)	73 (34.6)	
N1	62 (29.4)	60 (28.4)	
N2	43 (20.4)	43 (20.4)	
N3	46 (21.8)	33 (15.6)	
Nx or missing	8 (3.8)	2 (0.9)	
M			0.138^c^
M0	182 (86.3)	195 (92.4)	
M1	17 (8.1)	9 (4.3)	
Mx or missing	12 (5.7)	7 (3.3)	

^a^Data are presented as the number (%), AJCC: American Joint Committee on Cancer, T: tumor status, N: regional lymph node status, M: metastasis status.

^b^Data containing missing values were not included in the statistical analysis.

^c^Chi-square test with Yates' continuity correction.

### Validation of the RS signature in GEO datasets

**Figure 4:**
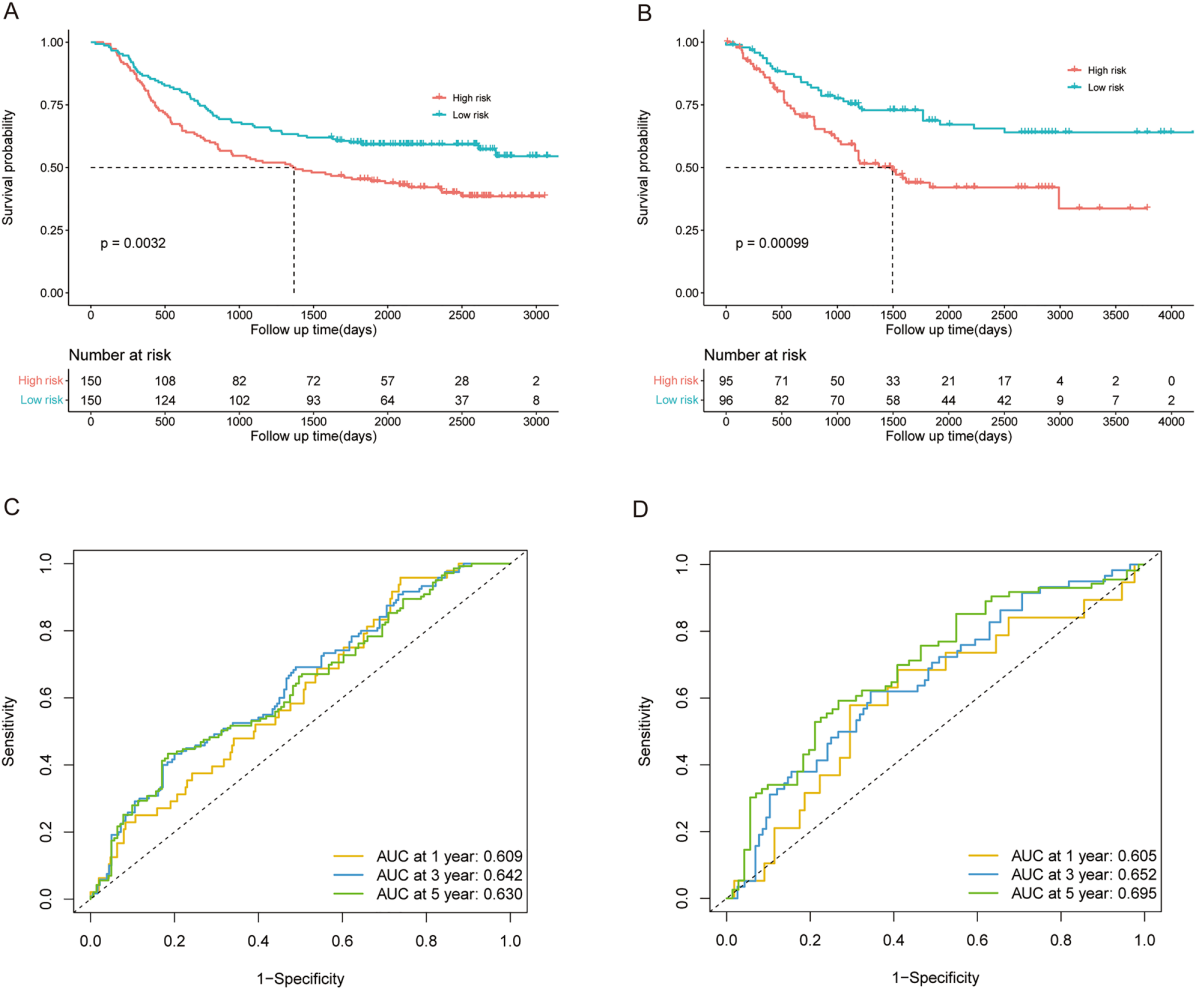
The prognostic value of the 5-genes RS signature in GEO validation datasets 1 (GSE62254) and 2 (GSE13861 and GSE26942). (**A**,**B**) Survival analysis of OS in patients with different RSs in the validation datasets 1 and 2. (**C**,**D**) ROC analysis of patients in validation datasets 1 and 2.

We used two independent validation datasets from the GEO database to assess the prognostic significance of this novel RS signature in patients with GC. With the risk score calculated by the Formula (2) mentioned above, the patients with GC in GSE62254 (validation dataset 1; n = 300) were divided into high risk (n = 150) and low risk (n = 150) subgroups according to the median risk score. Due to the limited sample size, we combined the GSE13861 and GSE26942 datasets as validation dataset 2 (n = 191), and the patients were also divided into high risk (n = 95) and low risk (n = 96) subgroups using the same methods mentioned before. Similar to the results found for the training datasets, the patients in the high risk subgroup tended to die earlier and have a significantly shorter survival time than did those in low risk subgroup in validation datasets 1 (*p* = 0.003, Fig. 4A) and 2 (*p* = 0.001, Fig. 4B). As shown in Fig. 4C,D, the AUC for validation datasets 1 and 2 reached 0.609 and 0.605 at 1 year, 0.642 and 0.652 at 3 years, and 0.630 and 0.695 at 5 years, respectively.

### Prognostic prediction in patients with different tumor stages

**Figure 5:**
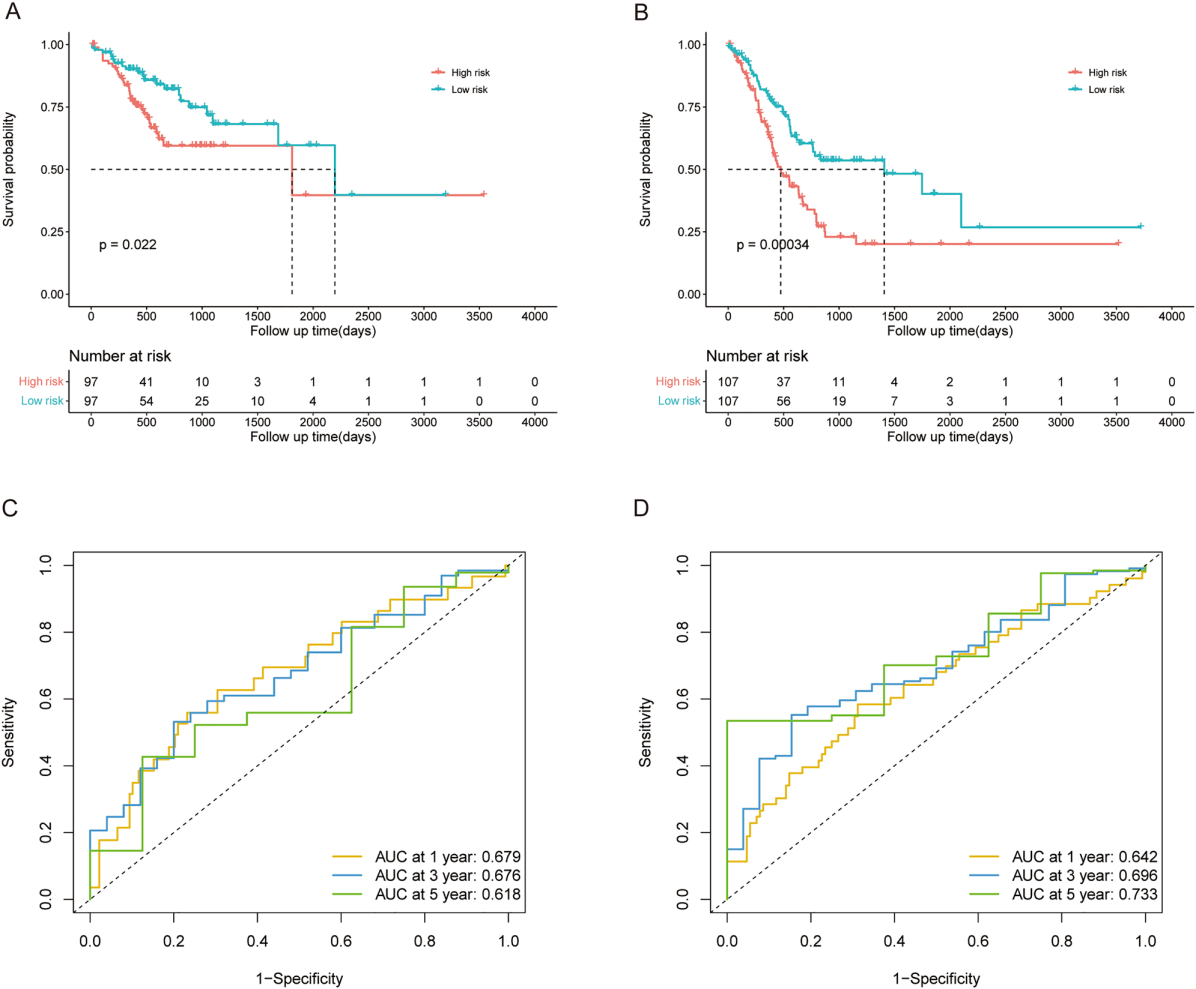
The predictive performance of the 5-genes RS signature in patients with different disease stages in the training cohort. (**A**) Survival analysis of OS in patients with different RSs and TNM stage I or II disease in the training dataset. (**B**) Survival analysis of OS in patients with different RSs and TNM stage III or IV disease in the training dataset. (**C**) ROC analysis of the RS in patients with TNM stage I or II disease in the training dataset. (**D**) ROC analysis of the RS in patients with TNM stage III or IV disease in the training dataset.

To further investigate the ability of the RS signature to predict OS, we applied KM survival analysis to OS in the training dataset based on patients with AJCC TNM stages I and II or III and IV. The RS signature showed an excellent predictive value for OS in patients with stage I or II disease (*p* = 0.022, Fig. 5A) or stage III and IV disease (*p* < 0.001, Fig. 5B). The AUC for the patients in stages I and II reached 0.679 at 1 year, 0.676 at 3 years, and 0.618 at 5 years (Fig. 5C), and it performed better in stage III and IV patients, with AUCs reaching 0.642, 0.696, and 0.733 at 1, 3, and 5 years, respectively (Fig. 5D).

### Independent prognostic value of the risk score

**Figure 6:**
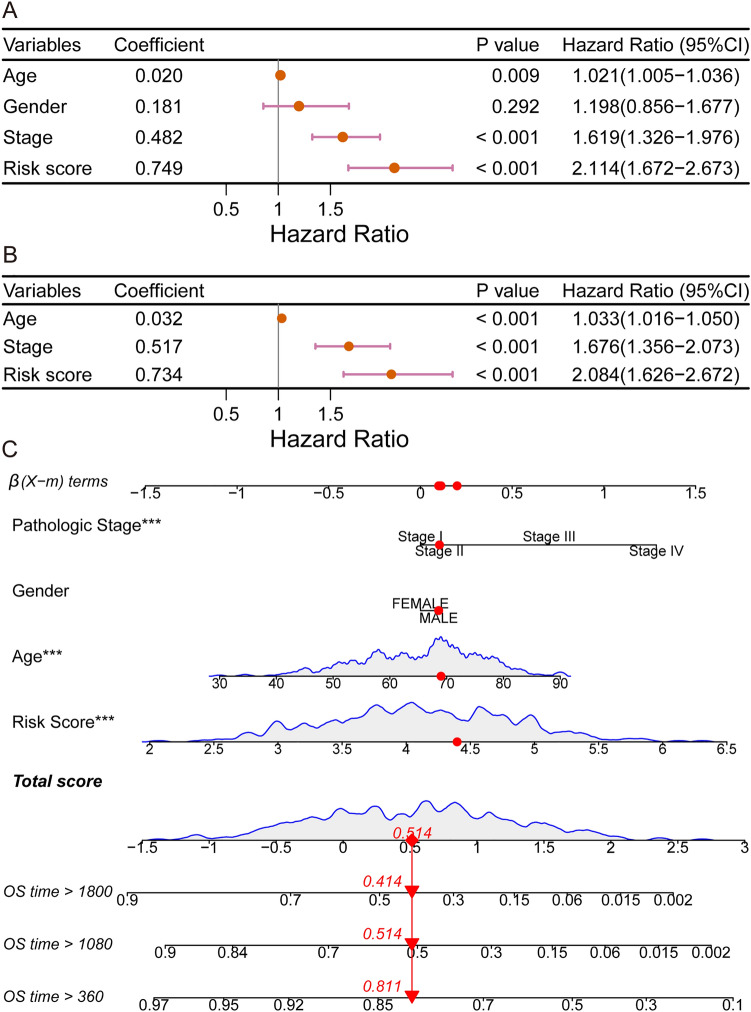
Comparison of the ability of the RS with other clinical indicators to predict the prognosis of GC patients (**A**,**B**) Results of univariate and multivariate Cox regression analyses in the training dataset. (**C**) Nomogram showing the performance of the AJCC TNM stage, sex, age, and RS in predicting the prognosis of GC patients according to multivariate Cox regression analysis.

We explored whether the risk score is an independent prognostic factor. In the training dataset, univariate Cox regression analyses showed that the risk score had a significant relationship with OS (hazard ratio (HR) = 2.114, 95% CI 1.672–2.673, *p* < 0.001; Fig. 6A) and a stronger predictive ability than other classical prognostic predictors, including age and the American Joint Committee on Cancer (AJCC) TNM stage. In multivariate Cox regression, risk score, age, and the AJCC TNM stage were evaluated for independent predictive capacity. The findings are shown in Fig. 6B. In terms of predictive ability, the risk score (HR = 2.084, 95% CI = 1.626–2.672, *p* < 0.001) was superior than age (HR = 1.033, 95% CI = 1.016–1.050, *p* < 0.001) and American Joint Committee on Cancer (AJCC) TNM stage (HR = 1.676, 95% CI = 1.356–2.073, *p* < 0.001). A nomogram containing the AJCC TNM stage, sex, age, and RS is presented in Fig. 6C.

### Favorable prognostic value of the risk score in different GC subtypes

**Figure 7:**
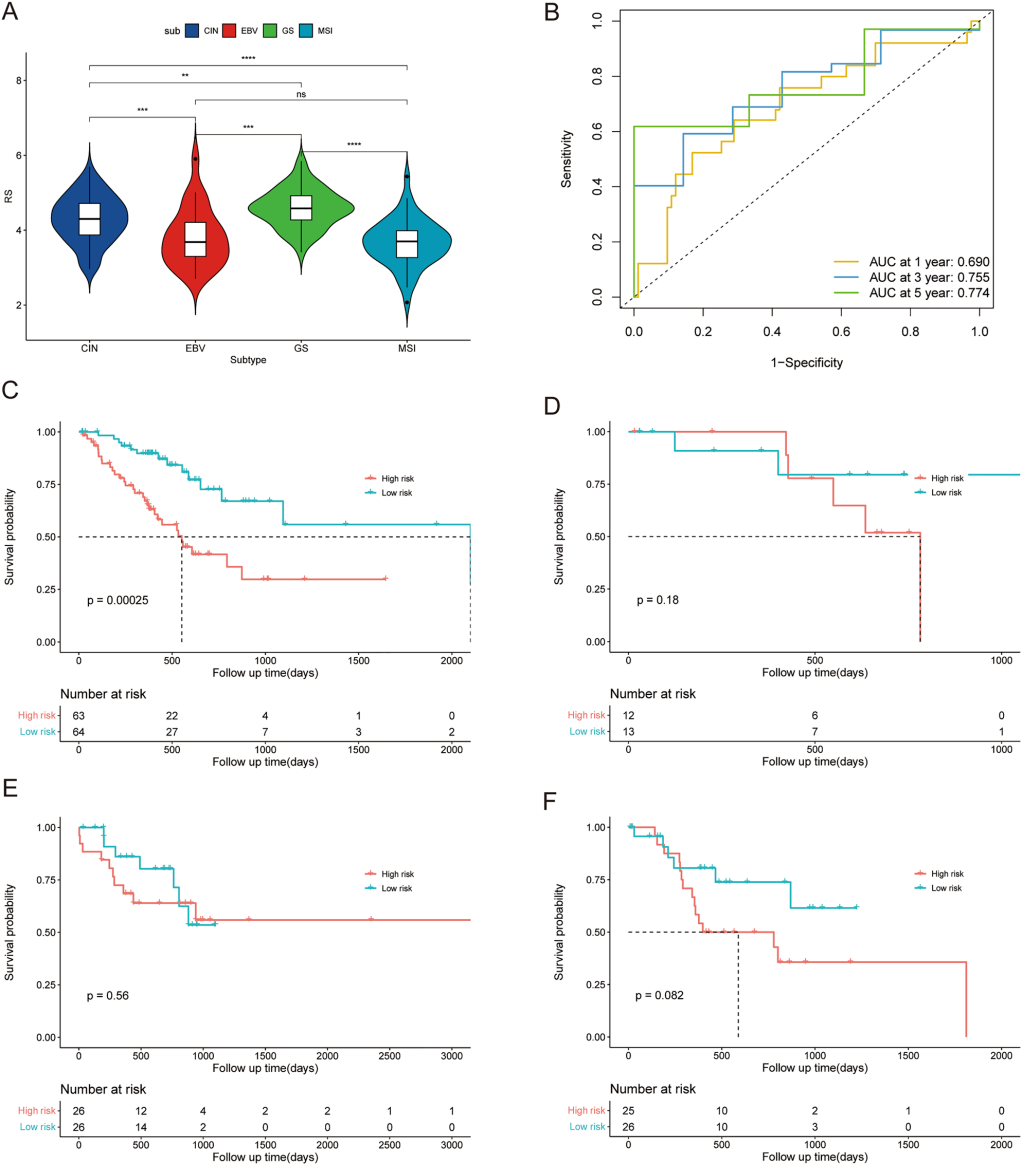
The predictive performance of the 5-gene signature in GC patients with different subtypes. (**A**) The distribution of the RS in patients with different GC subtypes in the training dataset. (**B**) ROC analysis of the RS in patients with the CIN subtype of GC in the training dataset. (**C**–**F**) Survival analysis of OS between patients with different RSs and those with four subtypes of GC in the training dataset. ns: p > 0.05, *: p ≤ 0.05, **: p ≤ 0.01, ***: p ≤ 0.001, ****: p ≤ 0.0001.

GC can be divided into 4 different molecular subtypes, CIN, EBV, GS, and MSI^26^. Figure 7A shows the risk score distribution in patients with different GC subtypes, which revealed higher RSs for GS and CIN, which are considered to have poorer prognoses than EBV and MSI^28,29^. Since each molecular subtype involves a different mutation, methylation, and immune signature^30^, we applied KM survival analysis of OS in the 4 different subtypes of patients in training datasets to further evaluate the prognostic value of the RS signature in GC patients with different subtypes. Figure 7B,C shows that the RS signature had good prognostic value for CIN (*p* < 0.001, n = 127) with an AUC reaching 0.690 at 1 year, 0.755 at 3 years, and 0.774 at 5 years. As the CIN subtype is considered to be related to poor prognosis in GC^29^, these results revealed the favorable prognostic value of the RS signature in patients with different GC subtypes. As shown in Fig. 7D–F, the RS signature had limited prognostic value for EBV (*p* = 0.180, n = 25), MSI (p = 0.560, n = 52), and GS (p = 0.082, n = 51). While the maximum sample size was only 52, the possibility could not be excluded that the sample size restricted the predictive ability of the RS signature.

### Functional enrichment analysis of genes screened out by multi-omics data

**Figure 8:**
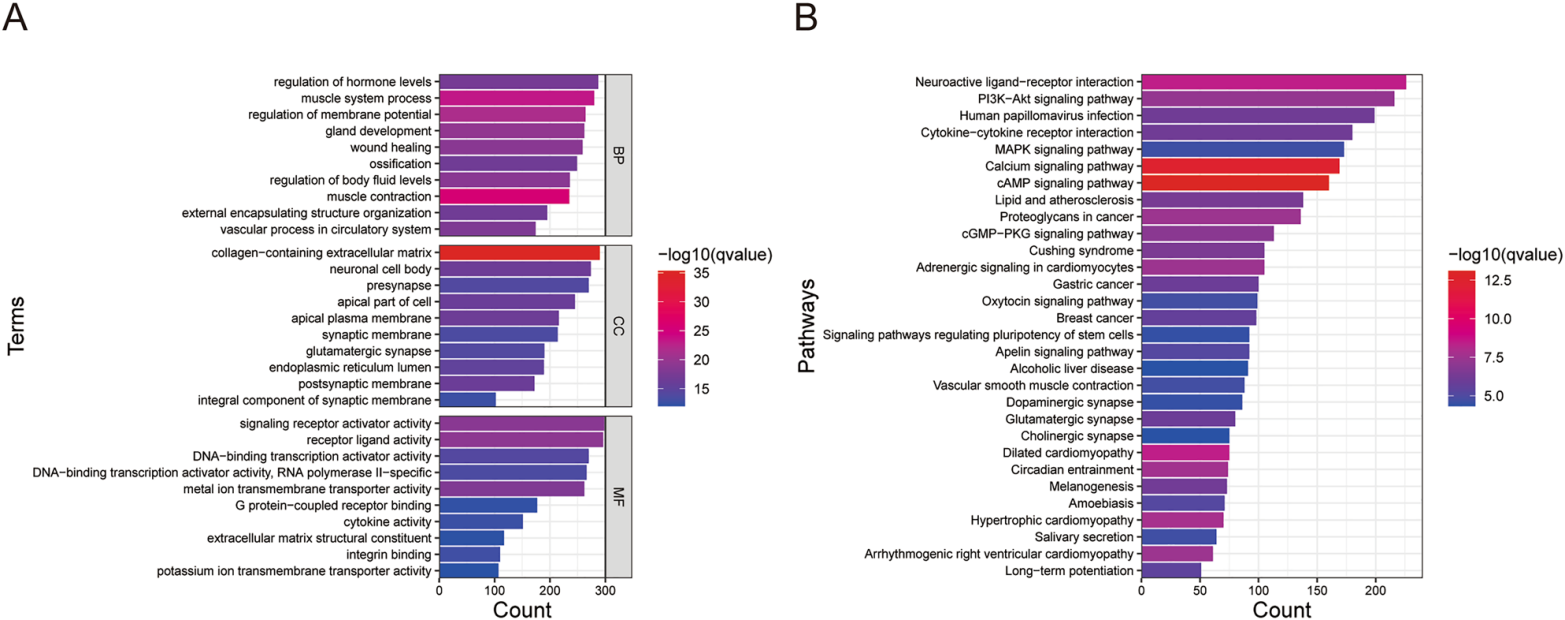
Functional enrichment analysis of genes screened out from multi-omics data from the training dataset in GC patients. (**A**) The results of GO analysis. (**B**) The results of KEGG enrichment analysis.

To clarify biological process (BP), cellular compartment (CC), molecular function (MF) terms and pathways correlating with the genes screened out by multi-omics data in the training dataset, enrichment analysis of GO terms and KEGG pathways was performed. According to GO enrichment analysis (Fig. 8A), the most enriched (sorted by *p* values) BP was muscle contraction, the most common CC was receptor ligand activity, and the most common MF was collagen-containing extracellular matrix. The top KEGG pathways (sorted by *p* value) related to the genes screened out by multi-omics data were the cAMP signaling pathway and calcium signaling pathway (Fig. 8B). These findings may indicate molecular changes in GC patients according to multi-omics data.

### Immune characteristics of patients with different risk scores

**Figure 9:**
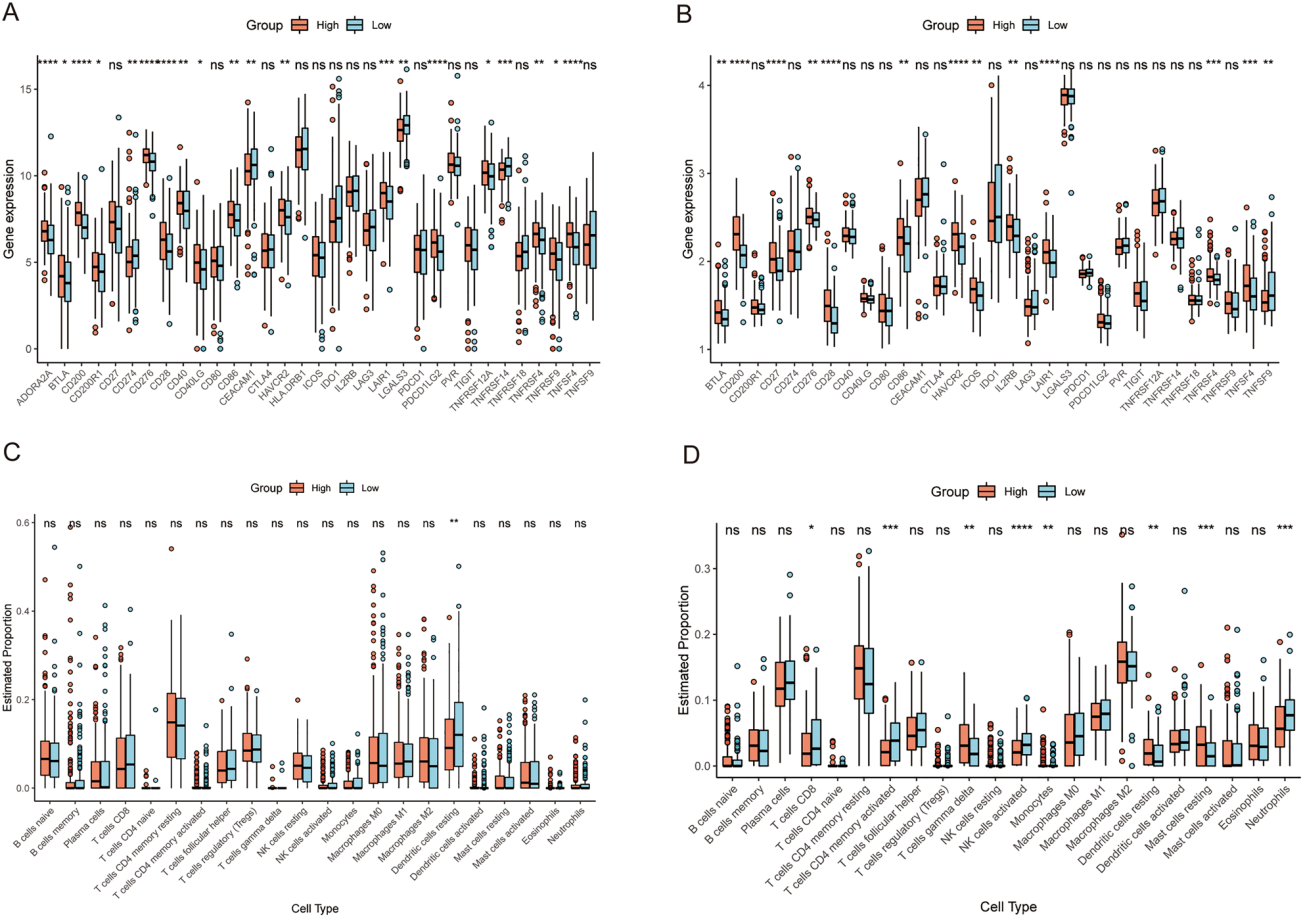
The immune characteristics of the 5-gene RS signature in the training dataset and GEO validation dataset 1 (GSE62254). (**A**,**B**) Expression of immune checkpoint genes among patients with different RSs in the training dataset and validation dataset 1. (**C**,**D**) Immune cell infiltration among patients with different RSs in the training dataset and validation dataset 1. ns: p > 0.05, *: p ≤ 0.05, **: p ≤ 0.01, ***: p ≤ 0.001, ****: p ≤ 0.0001.

We also compared immune characteristics between high risk subgroup and low risk subgroup, and the results are shown in Fig. 9. As shown in Fig. 9A, there were multiple immune checkpoint differences between the two high and low risk patient groups in the training cohort, but only the number of resting dendritic cells was significantly different between the two groups in immune infiltration analysis (Fig. 9C). However, in patients in validation dataset 1 from the GEO database, expression of several immune checkpoint genes and the proportions of several immune cells were altered (Fig. 9B and D). In both the training dataset and the validation dataset 1, expression of BTLA, CD200, CD28, CD86, HAVCR2, LAIR1, TNFRSF4, and TNFSF4 was upregulated in high risk patients, which indicated the association between the risk score and tumor immunity.

### Molecular docking

**Figure 10:**
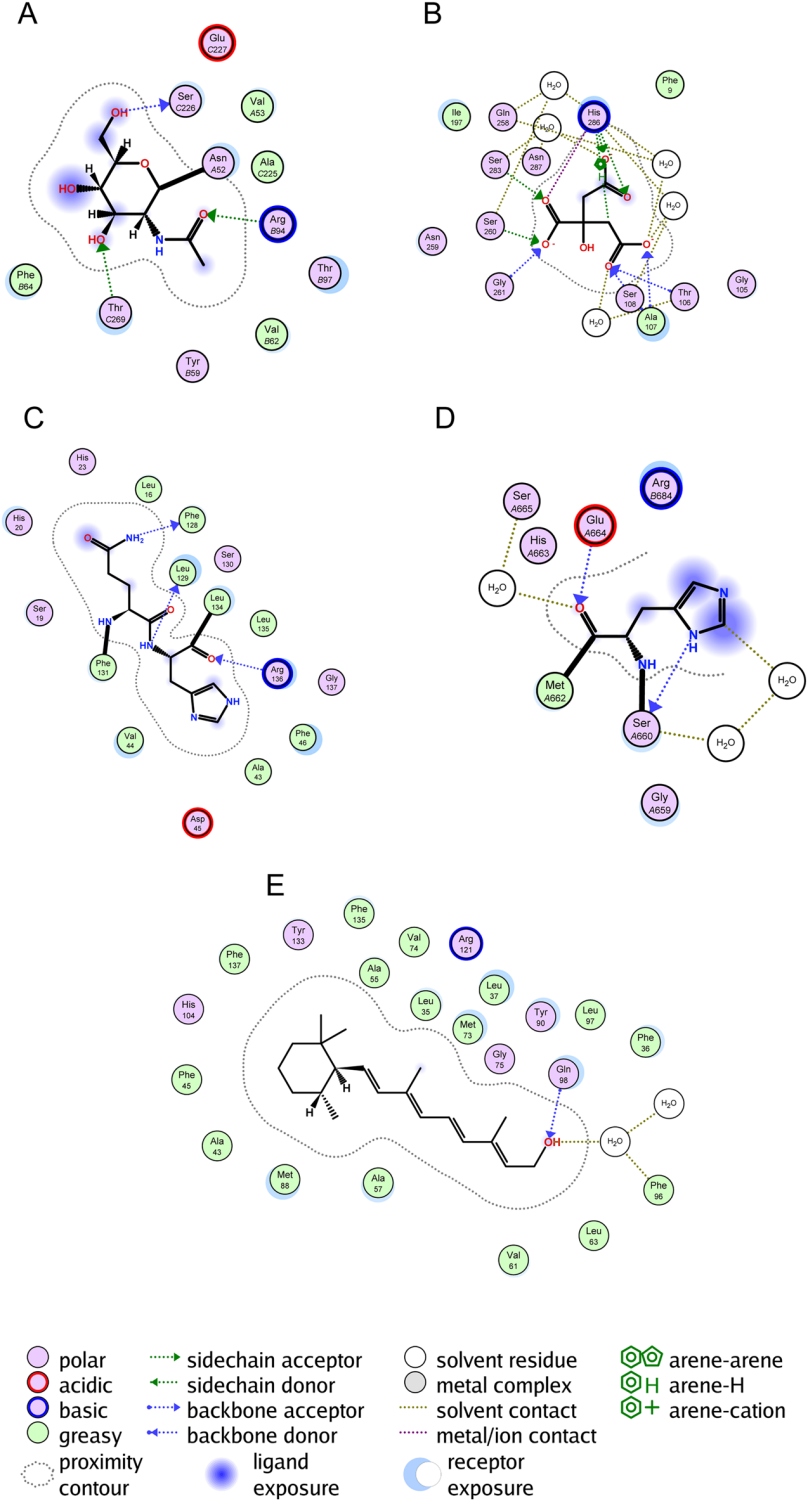
Molecular docking of docetaxel with (**A**) CGB5, (**B**) SLC10A2, (**C**) THPO. (**D**) PDGFRB, and (**E**) APOD.

Figure 10 shows that for the five constituent model genes, binding of the drug docetaxel differed significantly between patients in the high- and low-risk groups in the training dataset. CGB5 forms a side chain with Thr-C269 and Arg-B94. SLC10A2 forms a backbone with Ser108, Ala107, and Thr106. THPOs form backbones with Phe-128, Leu-129 and Arg-136. PDGFRB forms a backbone with Glu-A664 and Ser-A660. APODs form backbones with Gln-98 and solvent residues with Phe-96.

## Discussion

As one of the most prevalent cancers in the world, early detection of GC is problematic, and most patients are diagnosed at an advanced stage; even if they receive treatment, most patients experience recurrence or metastasis, resulting in poor prognosis and a 5-year survival rate of less than 30%^31^. Therefore, a signature that can accurately predict the prognosis of GC patient needs to be developed. Extensive study of multiple levels of biomolecules utilizing multi-omics is advantageous for exploring relationships among biological processes and is beneficial for determining the underlying mechanism in GC. The characteristics of single histology are insufficient for describing complex signaling pathways in organisms because the nature of life activities involves interaction of complex signaling pathways involving multiple molecules. Indeed, analysis of single-level molecules often omits essential information on physiological processes. In addition, molecules interact with each other at multiple levels in terms of the pathways and processes occurring in GC, which can increase the accuracy of data mining^32^.

In this study, we used TCGA-STAD gene expression RNA-Seq data, DNA methylation 27k array data, and gene-level CNV data, and integrated the significance of each gene using Fisher's method. Five genes strongly associated with the prognosis of GC patients were screened using univariate Cox regression analysis and LASSO regression analysis to construct the RS signature. The predictive power of the RS signature was subsequently validated using survival analysis and ROC curves analysis in a training dataset and two independent validation datasets. The results showed that the RS signature was effective at predicting prognosis of patients with GC. Patients classified by the RS signature into high risk subgroups in all three datasets had significantly worse survival probabilities than did those in the low risk subgroup (Figs. 3D,E, and 4). The following univariate and multivariate Cox regression analyses also showed that the RS signature correlated independently and significantly with GC patient prognosis (Fig. 6A,B). Among the four molecular subtypes of GC, patients with the EBV subtype had the lowest risk score, while patients with the GS subtype had the highest risk score (Fig. 7A). This conclusion is also consistent with previous research^28^, demonstrating that patients with the EBV subtype had the best prognosis and patients with the GS subtype the worst prognosis among the four molecular subtypes of GC. We then performed survival analysis and plotted ROC curves in the training dataset for patients with different disease stages and four MSs to verify the broad applicability of the RS signature. The results showed that the RS signature still had good predictive power for patients with different disease stages (Fig. 5A–D) and CIN subtypes (Fig. 7B,C) (with non-excessive sample size), demonstrating that the RS signature can effectively predict prognosis in a wide range of GC patient populations.

According to the results of functional enrichment analysis, the most enriched MF was the collagen-containing extracellular matrix (Fig. 8A). Collagens in the extracellular matrix act as ligands for immune inhibitory receptors^33^. One such receptor is LAIR1, which was more highly expressed in the high-risk subgroup than in the high-risk subgroup according to immune checkpoint analysis. LAIR1 signaling results in T-cell exhaustion and suppression and inhibition of natural killer, monocyte, and dendritic cell activation and function^34–36^, which reflects the intense immunosuppression in the high-risk subgroup and the predictive power of the RS signature for immune characteristics from another aspect. GO enrichment analysis also revealed enrichment of the regulation of membrane potential (Fig. 8A). Membrane potential can modulate critical cellular activities, which may impact tumor cell proliferation, migration, and differentiation^37,38^. Changes in membrane potential promote cell cycle checkpoint transition and are likely to trigger intracellular signaling messengers such as Ca^2+^ to drive sustained proliferation^37^. Moreover, the calcium signaling pathway was enriched according to KEGG enrichment analysis (Fig. 8B), which revealed some of the characteristics of the TME in GC patients. Furthermore, several terms or pathways related to signaling pathways, including receptor-ligand activity, signaling receptor activator activity, the cAMP signaling pathway, the PI3K-Akt signaling pathway, and the MAPK signaling pathway, were enriched according to GO and KEGG enrichment analyses. These findings reflect the complex signal transduction and immune regulation in the TME of GC. In summary, the enrichment landscape revealed by multi-omics data reflected several critical features of GC, providing clues for improving the treatment and prognosis of GC patients.

The immune characteristics of patients with different RSs were further examined. Immune checkpoints are several suppressive immune receptors/ligands that act as gatekeepers for the immune response^39,40^. In this study, we found that expression of 8 immune checkpoint genes, namely, BTLA, CD200, CD28, CD86, HAVCR2, LAIR1, TNFRSF4, and TNFSF4, was significantly increased in the high-risk subgroup in both TCGA and GEO cohorts (Fig. 9A,B).

BTLA is an inhibitory receptor belonging to the CD28 superfamily and a ligand of HVEM^17^. By preventing B and T-cell activation and proliferation, BTLA can cause immunosuppression. An increase in expression of BTLA and HVEM is considered to be associated with poor prognosis in GC patients^17,41^. A crucial costimulatory protein on the surface of T lymphocytes is CD28, which competes with other CD28 family members, such as CTLA-4, for binding to ligands of the B7 family, including CD80 and CD86^42^. In this study, we observed an increase in expression of CD86, a ligand for CD28. However, expression of CTLA-4, a competitive receptor of CD28, was not significantly different between the high- and low-risk subgroups, which may indicate a stronger CD28 costimulatory signal in the high-risk subgroup. CD28 costimulation is thought to enhance metabolic adaptation of tumor-infiltrating lymphocytes to restore metabolism and function in the TME^43–45^. However, the high-risk subgroup had poorer prognosis, which may reveal that other immune regulatory pathways inhibit the effect of CD28 co-stimulation. Successful checkpoint blockade treatment requires positive CD28 expression and co-stimulation^46–49^; a stronger co-stimulatory signal in patients with high risk scores may predict the effectiveness of immunotherapy.

There are several other immune checkpoint genes with altered expression. HAVCR2, often called TIM3, is highly expressed within the TME and correlates with suppression of T-cell responses and T-cell exhaustion, suggesting its role in tumor immunity^17,50,51^. The signal transduction generated by CD200 and its ligand CD200R is thought to regulate T-cell function, but its function in tumors is complex, and there is no consistent conclusion yet. LAIR1 is a kind of collagen domain-binding receptor^35^, that suppresses lymphocytic activity when binding to collagen, resulting in CD8^+^ T cell exhaustion and tumor immune suppression^52–54^. TNFRSF4 (OX40) and its ligand TNFSF4 (OX40L) are members of the TNFR/TNF superfamily^55^. Research has shown that there is increased expression of OX40 in GC patients while metastatic GC patients have higher soluble OX40 levels^56,57^; moreover, upregulated expression of OX40 is associated with better prognosis in such tumors^58,59^. Therefore, evaluating the relationship between GC prognosis and OX40 or OX40L is difficult. These findings of increased expression of immune checkpoint genes in the TCGA and GEO datasets demonstrated the high performance of the RS signature for risk-based grouping of GC patients in this study; the immune characteristics of the patients were well distinguished, providing information for treatment to achieve better prognosis.

Interestingly, the immune cell infiltration patterns of GC patients in the training and GEO (GSE62254) datasets were quite different (Fig. 9C,D). Among the American population in the TCGA cohort, only the proportion of resting dendritic cells was significantly greater in the low risk subgroup than in the high risk subgroup (Fig. 9C). As in the population from Korea in GSE62254, proportions of CD8 + T cells, activated CD4 + memory T cells, activated NK cells, and neutrophils were significantly greater in the low risk subgroup while those of gamma delta T cells, monocytes, resting dendritic cells, and resting mast cells were significantly lower in the low risk subgroup (Fig. 9D), revealing a stronger immune response in the low risk subgroup. We examined patient age in the two datasets to understand this difference. The results showed that the median (lower quartile, upper quartile) age of patients in the TCGA dataset was 67 (58, 74) years and that of patients in the GSE62254 was 64 years (55, 70). A rank sum test showed that the patient age in the TCGA dataset was greater than that in the GSE62254 dataset. We acknowledge that younger individuals usually have stronger immunity, which may partially explain the difference in immune cell infiltration. Studies have reported racial and ethnic differences in the incidence of GC worldwide and in America^60^, which suggests the influence of genetic background on GC and may also be the reason for the different results of immune infiltration analysis in populations from different regions. These results indicated that in patients from Korea, the different risk subgroups distinguished by our RS signature had distinct immune cell infiltration signatures.

The five hub genes that comprise the RS signature have been demonstrated in earlier research to be connected to the development of gastric or other cancers or to significantly impact patient prognosis. Overexpression of CGB5 in ovarian cancer cells results in increased receptor expression, and interaction between the two accelerates tumor growth and the development of ovarian cancer^61^. Sequence variants in SLC10A2 were observed to correlate with the risk of colorectal cancer^62^. Overexpression of THPO in gastric adenocarcinoma tumor tissues has been reported, and its high expression leads to poor prognosis^63^. PDGFRB affects GS metastasis and prognosis, and its co-expression with other genes is associated with reduced patient survival^64–66^. The prognosis of breast cancer patients is significantly impacted by APOD, which can be utilized as a biomarker^67–69^. These findings establish the relationship between the five genes that constitute the RS signature and cancer prognosis, and validate the RS signature in this study, which can be used to predict GC patient prognosis effectively. Molecular docking analysis reveals a strong binding affinity between docetaxel and the amino acid residues of PDGFRB and SLC10A2 proteins. The results of prior research findings also indicate that the products of these genes influence the action of the drugs. Inhibition of PDGFRB transcription has been found to be an important factor in docetaxel's effect on breast cancer^70^. The study by Deeken et al. found a correlation between SLC10A2 and docetaxel toxicity, which suggests the possibility that there is an association of this gene with docetaxel therapy, with potential implications for its efficacy^71^. Therefore, it suggests a potential therapeutic efficacy of docetaxel against GC. While the remaining genes also exhibit a binding affinity with docetaxel, the underlying mechanisms and precise impact remain contentious, warranting further research.

## Conclusion

In conclusion, this study used gene expression RNA-Seq, DNA methylation, and CNV data for gastric cancer patients in the TCGA cohort and Fisher’s test in combination with multi-omics data to screen for five immune-related genes with high prognostic relevance for GC patients and to construct an RS signature. The results illustrated that the RS can be used to predict the prognosis of GC patients effectively and is independent of other clinical indicators. The RS signature provides a new diagnostic approach and therapeutic target for GC, which might improve the prognosis of GC patients if validated by further experiments.

### Supplementary Information

Supplementary Information.

## Data Availability

The data used to support the findings of this study are available at UCSC (https://xenabrowser.net/datapages/) and GEO (http://www.ncbi.nlm.nih.gov/geo/) databases, accession numbers: GSE62254, GSE26942, and GSE13861.
